# METALS EXPOSURE AND COGNITIVE DECLINE IN LATINO ELDERS: RESULTS FROM THE SACRAMENTO AREA LATINO STUDY ON AGING

**DOI:** 10.1093/geroni/igac059.872

**Published:** 2022-12-20

**Authors:** Helen Meier, Kelly Bakulski, Allison Aiello

**Affiliations:** University of Michigan, Ann Arbor, Michigan, United States; University of Michigan School of Public Health, Ann Arbor, Michigan, United States; Carolina Population Center, University of North Carolina at Chapel Hill, Chapel Hill, North Carolina, United States

## Abstract

Latinos have a higher risk of Alzheimer’s disease and related dementias (ADRD) and clinical presentation could take place 7 years earlier, on average, than non-Hispanic whites. This health disparity will likely intensify as the US Latino population over 65 years is predicted to grow from 4 million in 2016 to 19.9 million in 2060. Environmental exposures, such as metals, are of interest as contextual factors contributing to ADRD due to their known neurotoxic effects, particularly in early life. Using data from the Sacramento Area Latino Study on Aging, we examined the association between blood concentrations of five metals and cognitive outcomes. We hypothesized that Latino elders with higher concentrations of non-essential metals will have greater cognitive decline than those with lower concentrations of non-essential metals. Initial results suggest non-essential blood metal levels are not sociodemographically patterned in SALSA participants. Higher lead and mercury concentrations was associated with cognitive decline.

